# High Molecular Weight (HMW): total adiponectin ratio is low in hiv-infected women receiving protease inhibitors

**DOI:** 10.1186/1472-6890-14-46

**Published:** 2014-12-16

**Authors:** Fierdoz Omar, Joel A Dave, Judy A King, Naomi S Levitt, Tahir S Pillay

**Affiliations:** Division of Chemical Pathology, C17 NHLS, Groote Schuur Hospital, University of Cape Town, Anzio Road Observatory, Cape Town, 7925 South Africa; Division of Diabetic Medicine and Endocrinology, Groote Schuur Hospital and University of Cape Town, Cape Town, South Africa; Department of Chemical Pathology, University of Pretoria and NHLS Tshwane Academic Division/Steve Biko Academic Hospital, Tshwane, South Africa

**Keywords:** HMW adiponectin, Lipodystrophy syndrome, HMW: adiponectin ratio, Protease inhibitors, Insulin resistance

## Abstract

**Background:**

At the time of the study, the HIV-treatment policy in South Africa included highly active antiretroviral therapy (HAART) regimens 1 (nucleotide reverse transcriptase inhibitors (NRTIs) only), and 2 (protease inhibitors (PI) and NRTIs). HAART is associated with the lipodystrophy syndrome, insulin resistance and reduced total adiponectin (TA) levels. The high molecular weight (HMW):TA ratio is a superior marker of insulin resistance. The aim of this study was to establish whether HMW:TA ratios are low in patients on PIs and whether they correlate with insulin resistance.

**Methods:**

This was a cross-sectional study undertaken in an antiretroviral clinic at a tertiary hospital. The participants were 66 HIV-infected females: 22 were on regimen 2 (PI group), 22 on regimen 1 (non-PI) and 22 treatment naïve (TN), matched for BMI and age. Patients with a history of diabetes or impaired glucose tolerance were excluded. Serum adiponectin multimers were analysed using the AlpcoTM Adiponectin (Multimeric) enzyme immunoassay. Waist hip ratios (WHR), glucose and insulin levels were assessed, and HOMA-IR and QUICKI calculated. Data were analysed non-parametrically and multivariate analysis was performed.

**Results:**

TA and HMW levels were lower in the treatment groups than in the TN group. HMW:TA was lower in the PI than in the non-PI and TN groups, and in the non-PI than in the TN groups. HMW:TA correlated negatively with waist, insulin and HOMA-IR, independently of BMI and duration of therapy. HOMA-IR and QUICKI did not differ among the groups.

**Conclusion:**

HMW:TA is significantly decreased with HAART (particularly with PIs, but also with non-PIs) and may be a more sensitive marker of insulin resistance in these patients than conventional markers or HMW and total adiponectin individually.

## Background

Adiponectin is an insulin-sensitising hormone found in multimeric forms in the circulation with the high molecular weight (HMW) 16-18mer (>400 kDa) being the predominant and active form [1].

Although an adipokine, unlike other hormones secreted by adipocytes, adiponectin levels are reduced in people with increased central body fat [1], insulin resistance, type 2 diabetes mellitus and atherosclerosis, as well as in individuals with lipoatrophy and lipohypertrophy [1]. HMW adiponectin has been shown to correlate better with insulin sensitivity than total adiponectin (TA) [2] and the HMW:TA ratio to be a better predictor of coronary artery disease than TA [3]. The ratio has also been shown to be suppressed in type 2 diabetes mellitus patients with coronary artery disease even when HMW and TA levels were unchanged [4].

In HIV-associated lipodystrophy, a syndrome consisting of fat redistribution, dyslipidaemia and insulin resistance, adiponectin levels are significantly lower, demonstrating a negative correlation with abdominal visceral fat mass and insulin resistance [5, 6]. This syndrome is associated with antiretroviral (ARV) therapy, particularly protease inhibitors (PIs) (but also nucleotide- and nucleoside reverse transcriptase inhibitors such as stavudine (d4T), zidovudine (AZT)) and didanosine (ddI) [7, 8]. In such patients, thiazolidinedione administration, via peroxisome proliferator-activated receptor γ activation, leads to improved insulin sensitivity [9] with upregulation of adiponectin levels, specifically the HMW form [10, 11]. Adiponectin administration in mice markedly ameliorates protease-induced dyslipidaemia, suggesting that hypoadiponectinaemia may be partially responsible for the metabolic derangements associated with PIs [12].

In South Africa, the National Department of Health had two highly active antiretroviral therapy (HAART) regimens at the time of this study. The first regimen consisted of d4T, lamivudine (3TC) and either efavirenz (EFV) or nevirapine i.e. a combination of two nucleotide reverse transcriptase inhibitors (NRTIs) and one non-nucleotide reverse transcriptase inhibitor (NNRTIs); while the second regimen consisted of AZT and lopinavir/ritonavir (LPV/r). AZT and ddI are nucleotide- and nucleoside reverse transcriptase inhibitors, respectively, while LPV/r is a PI.

The purpose of this study was to establish whether PI therapy was associated with lower HMW:TA ratios in HIV-infected patients, and to examine associated biochemical evidence of insulin resistance in these patients.

## Methods

This cross-sectional study was performed in accordance with the Helsinki Declaration. The protocol was approved by the University of Cape Town Faculty of Health Sciences Research Ethics Committee with reference number REC 450/2006. Sixty-six HIV-infected African females were enrolled into the study into three groups, viz. PI (Regimen 2 for at least six months), non-PI (Regimen 1 for at least six months) and treatment naïve (TN) groups, each consisting of 22 patients. Subjects were recruited from the ARV clinic at Groote Schuur Hospital, with the non-PI and TN groups matched to the PI group for body mass index (BMI) and age. Exclusion criteria included a history of impaired glucose tolerance or diabetes mellitus, active acute opportunistic infections, renal failure and pregnancy. Written informed consent was obtained. Waist and hip circumferences, weight and height were measured, and the BMI and waist: hip ratio (WHR) calculated. A 75 g OGTT was performed and blood samples drawn at 0 and 120 min. Glucose was measured at both time points and insulin and multimeric adiponectin in the 0 min sample only. Samples were centrifuged and stored at -70°C until analysis. The homeostatic model assessment for insulin resistance (HOMA-IR) and quantitative insulin-sensitivity check index (QUICKI) were calculated. Adiponectin was analysed using the Alpco™ Adiponectin (Multimeric) enzyme immunoassay (sensitivity 0.04 ng/mL and coefficient of variation (CV) <15%), insulin by the Bayer ACS180 auto-analyser (CV 12%), and glucose by the Bayer Alera chemistry analyser.

### Statistical analysis

Results were analysed non-parametrically, using the Mann–Whitney U, Kruskall-Wallis and Spearman correlation tests. Multivariate analyses and power calculations were performed. A p-value of 0.05 was considered significant.

## Results

### Patient characteristics

In the PI group, 21 patients received LPV/r and one Atazanavir for at least six months, with the median duration on PIs being 11.5 months. The median duration on regimen 1 drugs prior to progression to regimen 2 was 15 months. Four (18%) patients received d4T and 13 (59%) AZT, as part of their regimen. The median total duration on these drugs were 15.5 and nine months, respectively. In the non-PI group, 21 of the 22 patients received either AZT or d4T, with median durations of 12 and 10 months, respectively.

The median (interquartile range (IQR) ) age and BMI among the groups were 36 (29; 42) years and 27 (24; 30), respectively. Waist measurement and WHR did not differ among the groups (Table 1).

**Table 1:** **Adiponectin and markers of insulin resistance among the treatment groups (A), duration of therapy among the groups (B)**

*(A) Adiponectin and markers of insulin resistance among the treatment groups*					
	PI *Median (IQR)*	Non-PI *Median (IQR)*	TN *Median (IQR)*	Units	p-value
Age	34.5 (29.8; 41.0)	34.5 (31.3; 41.0)	35.5 (29.0; 41.0)	Years	0.986
BMI	27.7 (24.9; 30.4)	25.0 (23.8; 29.5)	25.7 (22.9; 29.7)	-	0.586
Waist	89 (82; 95)	83 (80; 93)	82 (76; 88)	cm	0.194
WHR	0.87 (0.82; 0.93)	0.85 (0.79; 0.89)	0.84 (0.80; 0.90)	-	0.315
Fasting insulin	5.5 (2.7; 10.1)	3.1 (2.1; 5.7)	4.4 (2.0; 8.0)	mIU/L	0.365
Fasting glucose	86 (81; 90)	91 (83; 98)	91 (85; 97)	mg/dL	0.145
2 hour glucose	104 (80; 113)	94 (85; 109)	94 (81; 109)	mg/dL	0.877
HOMA-IR	1.18 (0.54; 2.09)	0.72 (0.50; 1.17)	1.05 (0.41; 1.73)	-	0.479
QUICKI	0.37 (0.34; 0.42)	0.41 (0.37; 0.43)	0.38 (0.35; 0.45)	-	0.234
Total adiponectin	5.6 (3.4; 9.4)	7.3 (4.0; 8.7)	9.0 (7.6; 12.1)	ng/mL	0.039*
HMW adiponectin	2.2 (1.0; 3.9)	3.5 (1.8; 5.1)	5.3 (3.8; 7.0)	ng/mL	0.002*
HMW:Total adiponectin	0.35 (0.29; 0.42)	0.48 (0.41; 0.56)	0.56 (0.52; 0.61)	-	<0.0001**
**(B) Duration of therapy among the treatment groups**					
Regimen 1	15 (8.5; 21.5)	12 (9.5; 20.0)	-	Months	
Regimen 2	11.5 (9.3; 14.0)	-	-	Months	
d4T	15.5 (9.8; 22.3)	10 (8; 17.5)	-	Months	
AZT	9 (5; 16.5)	12 (11; 15.0)	-	Months	

The Kruskall-Wallis and Mann–Whitney U tests were used to assess differences among the groups collectively and individually, respectively.

PI, protease inhibitor group; Non-PI, non-protease inhibitor group; TN, treatment-negative group; IQR, interquartile range; HMW, high molecular weight. *p < 0.05; **p < 0.001. Multiply by the following conversion factor for SI units: glucose 0.0555 (mmol/L); insulin 6.945 (pmol/L).

### Adiponectin levels in serum (Figure 1 and Table 1)

**Figure 1:**
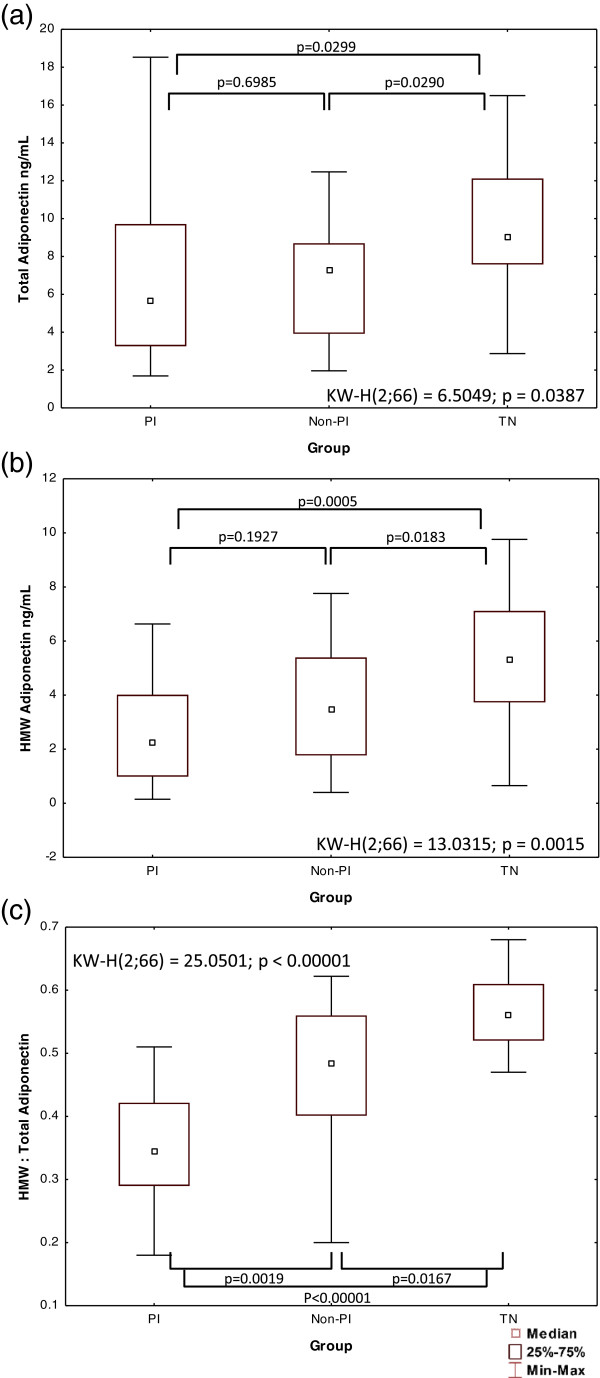
**Distribution of (a) Total adiponectin, (b) HMW adiponectin, and (c) HMW: total adiponectin ratio among the three groups.** HMW, high molecular weight; PI, protease inhibitor group; Non-PI, non-protease inhibitor group; TN, treatment-negative group; KW, Kruskall-Wallis; p <0.05 considered significant.

TA levels were within the reference interval (3.5 – 22 mg/L) [13] in all groups. However, total and HMW adiponectin levels were significantly lower in both treatment groups compared to the naïve group, with no significant difference between the PI and non-PI groups. In contrast, the HMW:TA ratio differed among all three groups and was significantly lower in the PI group than in both the non-PI and TN groups, and lower in the non-PI group than in the TN group. This difference was maintained when adjusting for BMI (data not shown) (Table 1 and Figure 1).

### Traditional markers of insulin resistance

There were no significant differences among the groups for insulin, fasting and 2-h glucose, and the derived parameters HOMA-IR and QUICKI (Table 1). However, 15 (23%) of the entire study sample of HIV-infected patients had insulin resistance, defined as HOMA-IR >1.95 [14], and their HMW:TA ratios were lower (p = 0.0059) (median 0.31, IQR 0.22 and 0.47) compared to those without insulin resistance (median 0.50, IQR 0.40 and 0.57). Similarly, 40 (60%) of the entire sample of HIV-infected patients were overweight (BMI ≥25) and had significantly lower (p = 0.0475) HMW:TA ratios (median 0.44, IQR 0.31 and 0.54) than those who were not overweight (median 0.50, IQR 0.44 and 0.58).

HMW, TA, and their ratio correlated negatively (p <0.05) with waist, BMI, fasting insulin and HOMA-IR, even when adjusted for BMI (Table 2). While HMW, TA, and their ratio correlated positively (p < 0.05) with QUICKI, this significance was lost when adjusting for BMI. When adjusting for both BMI and total duration of therapy, only the HMW:TA ratio maintained a significant correlation with fasted insulin, HOMA-IR and QUICKI, while all three adiponectin parameters remained significantly associated with waist. TA, HMW and their ratio maintained their association with waist, fasting insulin and HOMA-IR when adjusting for duration of therapy alone. HMW and HMW:TA also remained significantly associated with QUICKI when adjusting for duration of therapy alone.

**Table 2:** **The relationship between adiponectin and various markers of insulin resistance before and after adjusting for BMI and duration of therapy, separately and together**

*Relationship between adiponectin and markers of insulin resistance*												
		Univariable		Multivariable								
				*Adjusted for BMI*			*Adjusted for duration of therapy*			*Adjusted for BMI and duration of therapy*		
	b	95% CI	p-value	b	95% CI	p-value	b	95% CI	p-value	b	95% CI	p-value
**Total Adiponectin**												
Waist	-0.27	(-0.3895,-0.1420)	0.0001**	-0.28	(-0.5153,-0.0517)	0.019*	-0.29	(-0.4582,-0.1161)	0.002*	-0.35	(-0.6479,-0.0492)	0.028*
Fasted Insulin	-0.28	(-0.4683,-0.0906)	0.005*	-0.21	(-0.3945,-0.0164)	0.037*	-0.28	(-0.5408,-0.0266)	0.036*	-0.24	(-0.4935,0.0099)	0.067
HOMA-IR	-1.11	(-1.8677,-0.3428)	0.006*	-0.81	(-1.5741,-0.0551)	0.040*	-1.21	(-2.3196,-0.1036)	0.038*	-1.07	(-2.1435,0.0051)	0.058
QUICKI	14.20	(1.2607,27.1444)	0.035*	8.24	(-4.8207,21.3036)	0.221	15.25	(-0.2780,30.7806)	0.061	11.29	(-4.4271,26.9992)	0.167
**HMW Adiponectin**												
Waist	-0.17	(-0.2484,-0.0923)	0.0001**	-0.19	(-0.3350,-0.0429)	0.014*	-0.16	(-0.2637,-0.0627)	0.003*	-0.19	(-0.3674,-0.0109)	0.044*
Fasted Insulin	-0.17	(-0.2941,-0.0547)	0.006*	-0.13	(-0.2475,-0.0078)	0.041*	-0.17	(-0.3204,-0.0169)	0.035*	-0.14	(-0.2918,0.0048)	0.065
HOMA-IR	-0.69	(-1.1753,-0.2091)	0.007*	-0.51	(-0.9903,-0.0273)	0.042*	-0.72	(-1.3745,-0.0666)	0.037*	-0.64	(-1.2681,-0.0022)	0.056
QUICKI	9.38	(1.2388,17.5231)	0.027*	5.67	(-2.5563,13.9009)	0.182	9.97	(0.9112,19.0352)	0.037*	7.66	(-1.5098,16.8289)	0.110
**HMW: TA**												
Waist	-0.007	(-0.0102,-0.0042)	0.000**	-0.011	(-0.0165,-0.0054)	0.0003**	-0.16	(-0.2637,-0.0627)	0.003*	-0.007	(-0.0130,-0.0016)	0.016*
Fasted Insulin	-0.008	(-0.0123,-0.0030)	0.002*	-0.006	(-0.0109,-0.0014)	0.013*	-0.007	(-0.0119,-0.0025)	0.005*	-0.006	(-0.0111,-0.0018)	0.009*
HOMA-IR	-0.03	(-0.0497,-0.0123)	0.002*	-0.03	(-0.0442,-0.0063)	0.011*	-0.03	(-0.0509,-0.0103)	0.005*	-0.03	(-0.0479,-0.0084)	0.008*
QUICKI	0.42	(0.1036,0.7331)	0.011*	0.30	(-0.0230,0.6253)	0.073	0.44	(0.1586,0.7148)	0.004*	0.38	(0.0911,0.6598)	0.014*

HMW, high molecular weight; b, coefficient; CI, confidence interval. *p < 0.05; **p < 0.001.

## Discussion

Altered body composition and insulin resistance are components of the HIV–associated lipodystrophy syndrome [7]. This syndrome is primarily seen in HIV-infected patients on HAART, with a 17% risk of developing lipodystrophy after the first year of HAART and each additional 6 month period associated with a 45% risk [15]. These findings are particularly associated with PIs, but similar findings have been seen in some patients on nucleotide and nucleoside analogues such as d4T, AZT and ddI [8].

Adiponectin has been implicated in the pathogenesis of HIV– associated lipodystrophy [12], with PI administration in mice producing a dose-related reduction in adiponectin levels and administration of recombinant adiponectin ameliorating the associated dyslipidaemia.

It was therefore somewhat surprising that TA levels were well within the reference interval in all our study groups. However, they were significantly lower in the HIV-infected patients on HAART therapy (both PIs and non-PIs) than in those not receiving treatment.

The active HMW form of adiponectin has been shown to correlate better with insulin sensitivity than TA [16], and has been shown to be low in HIV-infected patients with insulin resistance [10]. In our study, HMW adiponectin levels were significantly lower in patients receiving both PIs and non-PIs, but also in those receiving only non-PIs. This finding in the latter group, may be attributed to the use of d4T (16 (66%) patients) or AZT (5 (23%) patients), drugs which have both been associated with lipodystrophy [17].

The HMW:TA ratio is a superior marker of insulin resistance compared to total and HMW adiponectin levels individually [18]. It is also an independent risk factor for coronary vascular disease (CVD) [19]. Previous studies have shown increased cardiovascular risk in HIV-infected patients, including those on HAART [20, 21]. We found this ratio to be significantly lower in the PI and less so (but nevertheless still significantly so) in the non-PI groups. This may imply an increased risk for CVD in patients on HAART (consistent with the findings of others) [20, 21], with the risk for CVD increased when both PIs and non-PIs are being used. This significantly lower HMW:TA ratio in patients receiving both PIs and NRTIs (with the majority, i.e. 17 of 22, of patients also receiving a thymidine analogue) confirms previous findings that protease inhibitors and thymidine analogues induce metabolic complications synergistically [7]. A limitation in this study is that the PI group also contained the nucleoside analogue ddI. Further studies are needed in the absence of so called d-drugs to confirm the contribution of PI drugs to the changes seen in this study.

Our patient groups were selected to exclude the presence of overt diabetes, and therefore it was not surprising that fasting glucose was not abnormal in any of the groups. On the other hand, we were surprised by the lack of difference in all measures of glucose tolerance and insulin sensitivity among the groups (Table 1), despite the difference seen among the groups in total and HMW adiponectin. However, a larger study sample may be required to verify this. The sample size was sufficiently powered (75.5%) to detect a difference in HMW: TA, but not to verify the lack of significant differences demonstrated for the insulin resistance markers. A further limitation is that visceral and subcutaneous adiposity were not quantitatively assessed. Notwithstanding, the relationship between insulin resistance markers and adiponectin, previously shown [5], was demonstrated here, with the HMW:TA ratio shown to be significantly lower in patients (in all groups) with established insulin resistance (HOMA-IR greater than 1.95 [21]) and also significantly lower in patients with BMI greater than or equal to 25 than in those with lower BMIs. Furthermore, adiponectin levels (high, total and their ratio) correlated negatively with HOMA-IR and positively with QUICKI. The HMW:TA ratio also correlated negatively with waist, waist hip ratio (data not shown), fasting insulin levels and BMI (data not shown) - all markers of insulin resistance. While BMI and duration of drug therapy collectively contribute to the association seen between the markers of insulin resistance and the TA and HMW adiponectin forms, they do not affect the associations seen with the HMW:TA ratio.

## Conclusion

These data demonstrate that both PI- and non-PI-containing HAART regimens significantly lower the HMW:TA ratio in HIV patients, with the ratio more significantly decreased in the PI-containing regimen, implying that PIs and NRTIs have an additive effect on the HMW:TA ratio. Although the HMW:TA ratio correlated negatively with indirect markers of insulin resistance, insulin resistance was not demonstrated to be associated with ARV drugs. We therefore propose that the HMW:TA ratio may be an earlier or more sensitive marker of insulin resistance in HIV-infected patients on HAART than conventional markers or HMW and total adiponectin individually.

## Authors’ information

FO: Chemical Pathologist; JD, Consultant Endocrinologist ; NL, Professor of Diabetes and Endocrinology; TSP: Professor and Chair, Chemical Pathology and Clinical Pathology.

## Abbreviations

HMW: High molecular weight
HIV: Human immunodeficiency virus
PI: Protease inhibitor
d4T: Stavudine
AZT: Zidovudine
HAART: Highly active antiretroviral therapy
3TC: Lamivudine
EFV: Efavirenz
ddI: Didanosine
NRTI: nucleotide reverse transcriptase inhibitors
NNRTI: non-nucleotide reverse transcriptase inhibitor
LPV/r: Liponavir/ritonavir
TA: Total adiponectin
ARV: Antiretroviral
non-PI: non-protease inhibitor
BMI: Body mass index
TN: Treatment naive
WHR: Waist: hip ratio
HOMA-IR: Homeostatic model assessment for insulin resistance
QUICKI: Quantitative insulin-sensitivity check index
CV: Coefficient of variation
IQR: interquartile range
CVD: coronary vascular disease
KW: Kruskall-Wallis.
